# Effects of noradrenaline and lipopolysaccharide exposure on mitochondrial respiration in alveolar macrophages

**DOI:** 10.1186/cc10627

**Published:** 2012-03-20

**Authors:** M Gröger, M Widman, J Matallo, P Radermacher, M Georgieff

**Affiliations:** 1 University of Ulm, Germany

## Introduction

Mitochondrial respiratory capacity of immune cells seems to be impaired in septic patients [1]. On the other hand, the effects of catecholamines on mitochondrial function are still controversial [2] and may confound the genuine mitochondrial response to the septic event. In order to test if catecholamine therapy may influence the impairment of mitochondrial function in immune cells during sepsis, we measured mitochondrial respiration in cultured murine alveolar macrophages (AMJ2-C11) after 24 hours of incubation with noradrenaline and lipopolysaccharide (LPS).

## Methods

Three states of mitochondrial respiratory activity were quantified in terms of O_2_-flux (JO_2_) in intact cells at 37°C by means of an O_2_K (Oroboros^® ^Instruments Corp., Innsbruck, Austria) according to a previously published protocol [3] yielding routine respiration (R) as the standard respiratory level of the cells without any intervention, proton leak compensation (L) after blocking ATP synthesis by 2.5 μM oligomycine, and maximum capacity of the electron transport system (E) after uncoupling by 1 μM FCCP. The cells were studied after five different exposure conditions: control (C), 15 μmol/ml noradrenaline (high NoA), 5 nmol/ml noradrenaline (medium NoA), LPS, and LPS + high NoA. All data are presented in pmol/(s*million cells) as medians and 25 to 75% quartiles. Statistical significance was tested by means of the Kruskal-Wallis one-way ANOVA followed by Dunn's method.

## Results

After exposure with high but not with medium NoA we observed a statistically significant decrease in maximum mitochondrial respiratory capacity (E-state, C 133 (118; 148) vs. high NoA 111 (106; 113), and medium NoA 129 (123; 140), *P *< 0.05 C vs. high NoA). Both LPS and LPS + high NoA did not affect E-state respiration (LPS: 152 (136; 179), and LPS + NoA 129 (125; 137)), but increased routine (R) respiration when compared to control (C 45 (40; 55) vs. LPS 66 (51; 72) and LPS + NoA 65 (55; 68), *P *< 0.05; high NoA 41 (37; 47), and medium NoA 52 (51; 57), NS).

## Conclusion

High but not moderate doses of noradrenaline reduced mitochondrial respiration in alveolar macrophages *in vitro*. Surprisingly, LPS increased routine respiration regardless of simultaneous noradrenaline exposure.
